# TCGA-My: A Systematic Repository for Systems Biology of Malaysian Colorectal Cancer

**DOI:** 10.3390/life12060772

**Published:** 2022-05-24

**Authors:** Mohd Amin Azuwar, Nor Azlan Nor Muhammad, Nor Afiqah-Aleng, Nurul-Syakima Ab Mutalib, Najwa Farhah Md. Yusof, Ryia Illani Mohd Yunos, Muhiddin Ishak, Sazuita Saidin, Isa Mohamed Rose, Ismail Sagap, Luqman Mazlan, Zairul Azwan Mohd Azman, Musalmah Mazlan, Sharaniza Ab Rahim, Wan Zurinah Wan Ngah, Sheila Nathan, Nurul Azmir Amir Hashim, Zeti-Azura Mohamed-Hussein, Rahman Jamal

**Affiliations:** 1 Center for Bioinformatics Research, Institute of Systems Biology (INBIOSIS), Universiti Kebangsaan Malaysia, UKM, Bangi 43600, Malaysia; m_aminazuwar@siswa.ukm.edu.my (M.A.A.); norazlannm@ukm.edu.my (N.A.N.M.); 2 Institute of Marine Biotechnology, Universiti Malaysia Terengganu, Kuala Nerus 21030, Malaysia; afiqahaleng@umt.edu.my; 3 UKM Medical Molecular Biology Institute (UMBI), Universiti Kebangsaan Malaysia, Kuala Lumpur 56000, Malaysia; syakima@ppukm.ukm.edu.my (N.-S.A.M.); najwa.fmy@gmail.com (N.F.M.Y.); ryia.yunos@ppukm.ukm.edu.my (R.I.M.Y.); muhiddin@ppukm.ukm.edu.my (M.I.); sazuita@ukm.edu.my (S.S.); rahmanj@ppukm.ukm.edu.my (R.J.); 4 Department of Pathology, Faculty of Medicine, Universiti Kebangsaan Malaysia, Jalan Yaacob Latif, Cheras, Kuala Lumpur 56000, Malaysia; isa@ppukm.ukm.edu.my; 5 Department of Surgery, Faculty of Medicine, Universiti Kebangsaan Malaysia, Jalan Yaacob Latif, Cheras, Kuala Lumpur 56000, Malaysia; ismailsagap@ppukm.ukm.edu.my (I.S.); luqman@ppukm.ukm.edu.my (L.M.); zairulazwan@ppukm.ukm.edu.my (Z.A.M.A.); 6 Department of Biochemistry and Molecular Medicine, Faculty of Medicine, Universiti Teknologi MARA, Campus Sungai Buloh, Sungai Buloh 47000, Malaysia; musalmah6393@uitm.edu.my (M.M.); sharaniza_abrahim@uitm.edu.my (S.A.R.); ask_me_2706@yahoo.com (N.A.A.H.); 7 Department of Biochemistry, Faculty of Medicine, Universiti Kebangsaan Malaysia Medical Centre, Kuala Lumpur 56000, Malaysia; wwanzurinah@yahoo.com; 8 Department of Biosciences and Biotechnology, Faculty of Science and Technology, Universiti Kebangsaan Malaysia, UKM, Bangi 43600, Malaysia; sheila@ukm.edu.my; 9 Department of Applied Physics, Faculty of Science and Technology, Universiti Kebangsaan Malaysia, UKM, Bangi 43600, Malaysia

**Keywords:** colorectal cancer, CRC database, CRC repository, TCGA-My, genome, metabolome, systematic repository

## Abstract

Colorectal cancer (CRC) ranks second among the most commonly occurring cancers in Malaysia, and unfortunately, its pathobiology remains unknown. CRC pathobiology can be understood in detail with the implementation of omics technology that is able to generate vast amounts of molecular data. The generation of omics data has introduced a new challenge for data organization. Therefore, a knowledge-based repository, namely TCGA-My, was developed to systematically store and organize CRC omics data for Malaysian patients. TCGA-My stores the genome and metabolome of Malaysian CRC patients. The genome and metabolome datasets were organized using a Python module, pandas. The variants and metabolites were first annotated with their biological information using gene ontologies (GOs) vocabulary. The TCGA-My relational database was then built using HeidiSQL PorTable 9.4.0.512, and Laravel was used to design the web interface. Currently, TCGA-My stores 1,517,841 variants, 23,695 genes, and 167,451 metabolites from the samples of 50 CRC patients. Data entries can be accessed via search and browse menus. TCGA-My aims to offer effective and systematic omics data management, allowing it to become the main resource for Malaysian CRC research, particularly in the context of biomarker identification for precision medicine.

## 1. Introduction

Globally, colorectal cancer (CRC) is the second leading cause of cancer death in both males and females. In 2020, there were an estimated 935,000 mortalities from CRC, which accounted for over 1.9 million cases [1]. CRC has become a major public health concern in the Asia-Pacific region, including Malaysia [2]. Malaysia is a developing country located in Southeast Asia with an estimated population of 32.7 million in 2021 and an annual population growth rate of 0.2%. The Malaysian National Cancer Registry Report 2012–2016 found that CRC is the most common cancer in males (16.9% of all cancers diagnosed) and the second most common cancer in females (10.7% of all cancers diagnosed) [3]. A study of the National Cancer Registry for CRC from 2008 to 2013 revealed that the overall age-standardized incidence rate for CRC was 21.32 per 100,000 population. Those of Chinese ethnicity had the highest CRC incidence rate (27.35), followed by Malay (18.95) and Indian (17.55) [4]. Despite the severity caused by CRC, local studies pertaining to this field are scarce. The first study on the local CRC omics has been conducted on 50 CRC patients from Hospital Canselor Tunku Muhriz, Cheras, Kuala Lumpur. Data from the 50 samples was used as a model for CRC database development, and it will also guide the next experiments on what type of data/samples will be needed to ensure the development of a comprehensive repository. In addition, TCGA-My was developed with the aim of providing a repository platform for a Malaysian cancer consortium. Cancer databases have been developed and curated using bioinformatics with the support of highly advanced experimental evidence to assist the discovery of novel and unknown information about cancer.

Even though there is a growing trend in CRC incidence and mortality, the pathobiology of CRC remains unknown [1,3], hence the need for a CRC omics database that will provide pools of genes, proteins or metabolites that are associated with this cancer, which can be identified through integrative analysis. To this end, genomic and metabolomic data was collected from 50 Malaysian CRC patients, and a database was developed to provide a systematic data storage and retrieval platform, which is known as Malaysian TCGA (TCGA-My). TCGA-My stores CRC data for the Malaysian ethnic population, and this feature distinguishes TCGA-My from other colorectal cancer databases, such as Colorectal Cancer Atlas (http://www.colonatlas.org (accessed on 10 May 2022)) [5], Colorectal Cancer Biomarker Database (CBD) (http://sysbio.suda.edu.cn/CBD/ (accessed on 10 May 2022)) [6] and Colon Rectal Cancer Gene Database (CoReCG) (https://lms.snu.edu.in/corecg/ (accessed on 10 May 2022)) [7], which store the genes or proteins associated with CRC regardless of the patients’ region of origin.

In TCGA-My, most of the genes and metabolites are fully annotated to help users explore functional information for the data. Biological information for chromosomes, DNA region, tissue occurrence and type of mutation for each variant are also included. Circos plots are generated and provided to help users visualize the variants on human chromosomes. Additionally, details for the samples such as gender, age and ethnicity are also provided as they are useful for the interpretation of various analyses of the data. Eventually, TCGA-My will provide CRC raw data and results from the integrative analysis that can be used to predict phenotypic changes in cancer cells upon chemo-immunotherapy treatment toward precision and personalized medicine initiatives. With these features, TCGA-My should serve as a comprehensive database that will be an excellent aid in providing and integrating accurate molecular information to understand the relationship between CRC and ethnicity.

We envision TCGA-My will expand and serve as a Malaysian cancer multi-omics data repository and provide a unique opportunity for a systems biology approach to tackle the complexity of cancer cells and CRC pathobiology through the unification of experimental data and computational/mathematical models. Currently, TCGA-My stores 1,517,841 variants, 23,695 genes and 167,451 metabolites obtained from 13 genomic and 50 metabolomic samples. Genomic experiments with another 37 samples are in progress and the data will be gradually deposited into this database. We used the available data to design the TCGA-My architecture. The development of TCGA-My is ongoing and accessible at https://tcgamy.inbiosis.org/(TCGA-My v1.0, last updated April 2022) (accessed on 10 May 2022).

## 2. Materials and Methods

### 2.1. Data Collection and Organization

The CRC genome data for 13 patients were obtained from Universiti Kebangsaan Malaysia Medical Molecular Biology Institute (UMBI). The Gene Analysis Tool Kit (GATK) was used to perform variant calling. ANNOVAR [8] was then used to annotate the variant with the associated gene. The samples from 13 patients were used as a test set to guide the CRC database development. As new data are generated, the database will be extended accordingly. The new data will also guide the choice of data/samples needed to ensure comprehensive repository coverage.

The metabolome data were obtained from the serum of 50 healthy controls and 50 CRC patients collected at UKM Medical Centre. Data analysis was conducted as described by Amir Hashim et al. [9]. 

Additional information on the individual molecules was obtained from external databases; for example, variants were linked to a dbSNP identifier (ID) [10] and COSMIC v70 ID [11]; genes were compared against GeneCards [12], Protein Databank (PDB) [13], RefSeq ncRNA [14] and UniProt [15]; metabolites were identified using an ID browser from Mass Profiler Professional software for the Metlin database [9]. Twenty-three CRC driver genes were identified by Abdullah and Muhammad [16]. The metabolites were also linked to Kyoto Encyclopedia Genes and Genomes (KEGG) ID [17]. CRC staging of the samples were compared against the Cancer Research UK website (https://www.cancerresearchuk.org (accessed on 1 March 2022)).

The data were organized using pandas, which is a Python module [18]. All variants for each sample were mapped onto a Circos plot. This plot was constructed using a protocol from Strawberry Perl [19].

### 2.2. Functional Annotation

Annotation was conducted to better understand the function of CRC sequences and metabolites. This was conducted using a standard bioinformatic procedure where extensive information on the sequences such as chromosomal location, gene ontology (GO) and pathway were retrieved from online databases such as NCBI Gene [20], Gene Ontology Consortium [21], KEGG [17], BioCarta [22], WikiPathways [23], InterPro [24], Human Protein Atlas [25], DisGeNET [26] and PANTHER [27], or were obtained from our bioinformatics analysis (where necessary).

### 2.3. Data Normalization

**Figure 1:**
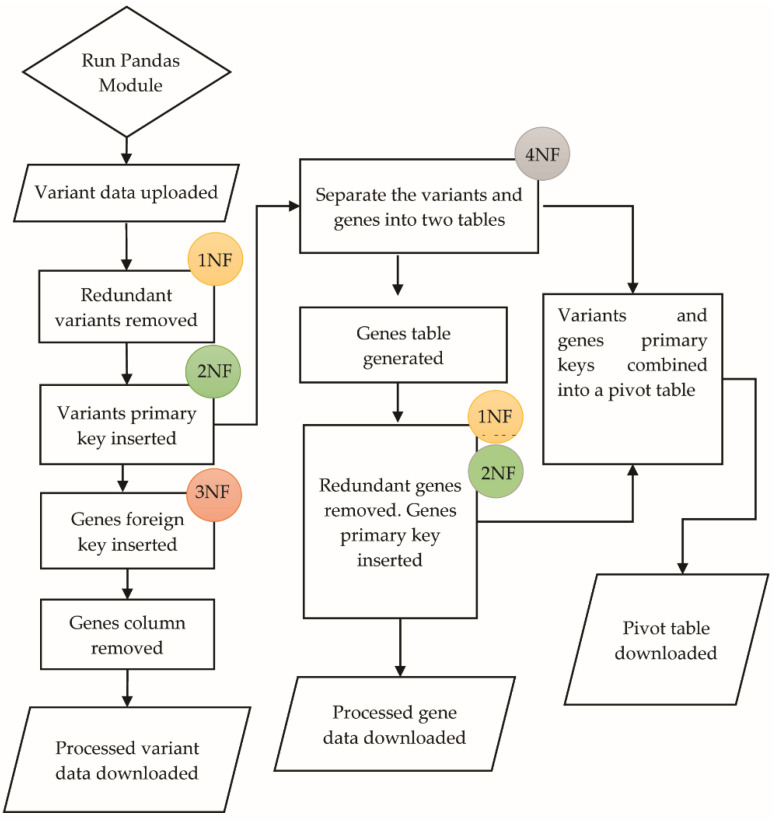
The variants and genes data normalization algorithm that was deployed for generating an SQL file for TCGA-My.

The repetition of the variant data was minimized by deploying four levels of data normalization [28] on the annotated variant dataset (Figure 1) as follows:

∘ 1NF: First Normal Form—Removal of redundant variants/genes data. The ANNOVAR output has the same variants for multiple genes and patients.
∘ 2NF: Second Normal Form—Insertion of variants/genes primary keys. Each unique variant and gene obtained its own primary key.
∘ 3NF: Third Normal Form—Insertion of foreign keys. The information and links between the variants and genes were converted into foreign keys.
∘ 4NF: Forth Normal Form—Separation of variants and genes data into separate tables. The primary keys for variants and genes were transferred into a pivot table.

### 2.4. Database Organization and Architecture

All collected data, including relevant information on the variants, genes and metabolites with their functional information were organized in 19 tables. Two types of tables were designed, which were (1) main tables that contained the collected data and (2) pivot tables that were linked with the main tables. TCGA-My was built as a relational database using HeidiSQL PorTable 9.4.0.512. The web interfaces were designed using a PHP web framework, Laravel.

## 3. Results

### 3.1. Database Summary

**Figure 2:**
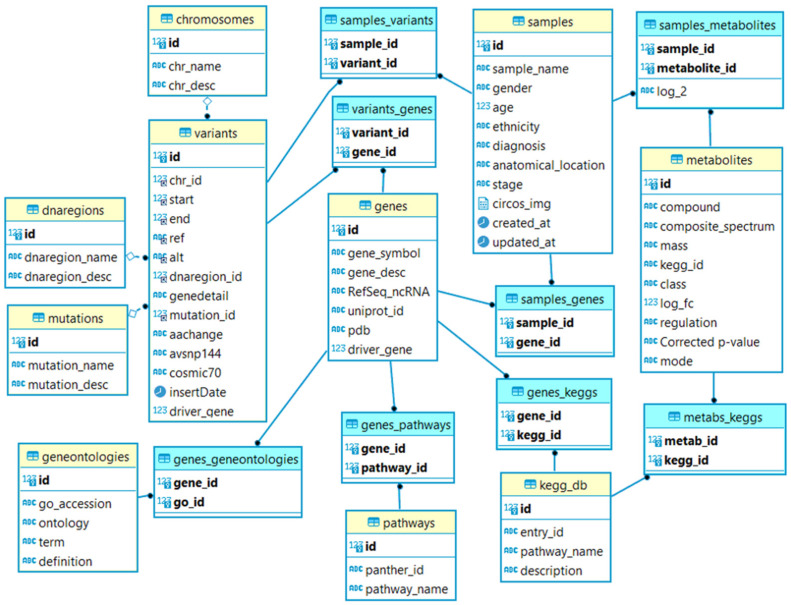
The relational tables for the TCGA-My schema. The yellow tables are the main tables, and the blue tables are the pivot tables. The relationship between main and pivot tables is indicated with a straight line (**―**●). The dotted line represents the relationship between main tables (◊---●).

Figure 2 illustrates the organization of four datasets, i.e., sample; variants with an ID obtained from COSMIC and dbSNP databases; genes with an ID retrieved from PDB, RefSeq ncRNA and UniProt; and metabolites. Table 1 summarizes the number of entries for each dataset.

### 3.2. Main Datasets

#### 3.2.1. Samples

Each sample of CRC represents data from different patients. Each sample was provided with the patients’ details, such as gender, age, ethnicity, diagnosis information, anatomical location and cancer stage (Table 2). Two cancer staging systems were included in this database, i.e., TNM [29] and Dukes staging [30] (Table 3).

#### 3.2.2. Variants

**Figure 3:**
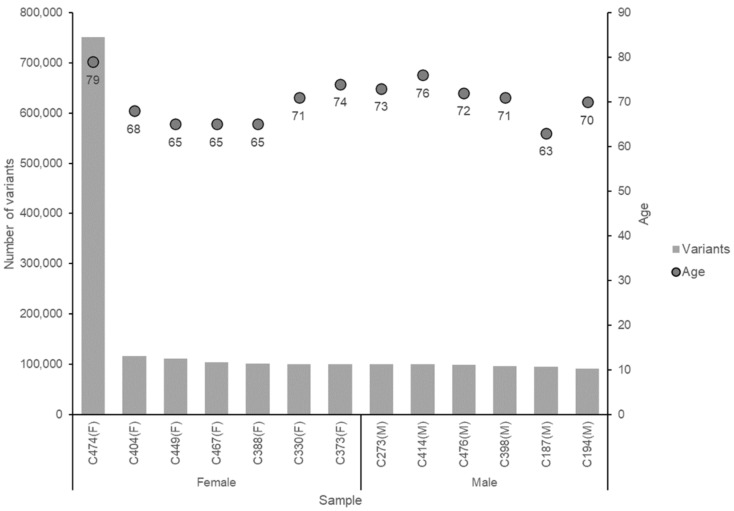
The correlation between the number of variants with the age and gender of CRC patients.

TCGA-My provides a list of variants with comprehensive mutation details that includes chromosomal location, variant start and end point, reference and alternate nucleotide, variant position in DNA region, affected gene, tissue occurrence and type of mutation caused by the variant. Figure 3 shows the correlation between the number of variants, gender and age of the CRC patients. The number of variants in 12 samples showed slight differences with a range of 95,000± to 116,000± variants. Only one sample (C474), a female with the oldest age of 79, showed the highest number of variants (751,000± variants). Age might influence the number of variants [31].

**Figure 4:**
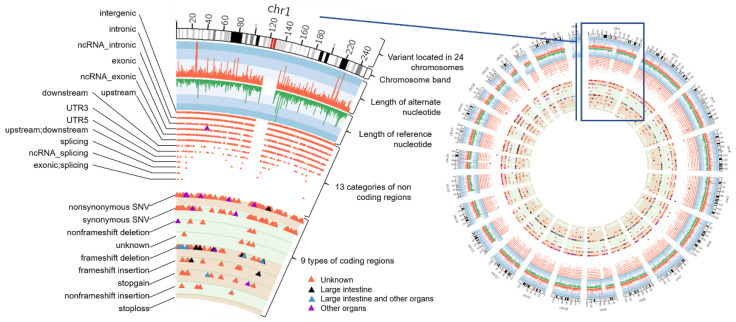
Circos plot for sample C474. The plot was constructed using Strawberry Perl to visualize the location of variants in the chromosomes.

Table 4 and Table 5 describe the position of variants in DNA regions and mutations in genome data, respectively, for 13 CRC patients. Circos plots display the position of the variants on 24 human chromosomes and the mutation types in a sample. A Circos plot was constructed for every patient and Figure 4 shows an example of the plot constructed for patient C474.

#### 3.2.3. Genes

The variant genes were provided with gene symbol descriptions and linked with three different IDs from ncRNA, PDB and UniProt IDs. Forty-four genes were recently annotated as CRC driver genes by Abdullah and Muhammad [16], where a computational approach was applied to identify CRC driver genes using a bioinformatics pipeline that consisted of the Cancer Genome Interpreter (CGI) [32] and Integrated Cancer Genome Score (iCAGES) [33] analysis platforms. Driver genes refer to genes whose mutations promote tumor growth [34], and further investigation of these genes is critical in precision oncology [35]. Table 6 lists the number of genes and driver genes for each patient.

#### 3.2.4. Metabolites

**Figure 5:**
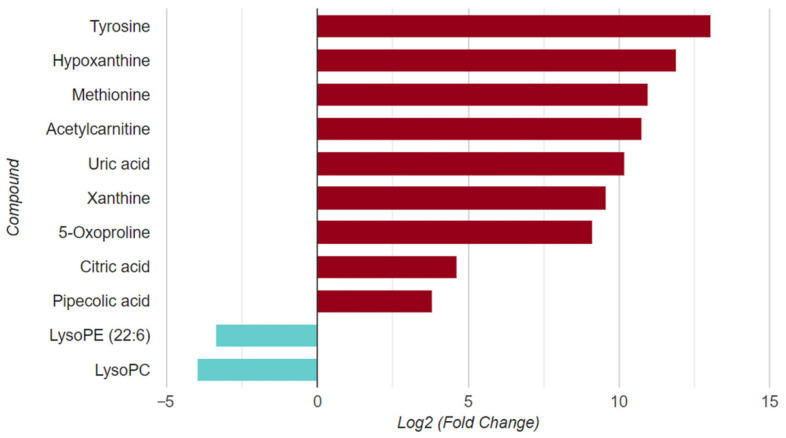
Significantly altered metabolites. The CRC metabolome samples reveal nine upregulated (red) and two downregulated (blue) metabolites.

The metabolites in TCGA-My were described with the name, class and mass. From 89,256 identified metabolites, only eleven metabolites were significantly altered, suggesting their potential as biomarkers for CRC (Figure 5).

### 3.3. Database Interface and Access

The TCGA-My interface contains four main menus, i.e., About, Browse, Search and Help, that were designed to help users easily navigate the respective pages. Each primary page has a header with a logo, search box and tabs that can be used to navigate to the primary pages and the dropdown menu. The logo on the header can be clicked to return to the homepage and the search box serves as the homepage search box.

- Homepage displays statistics for the main datasets, i.e., sample, variant, gene and metabolites. The number of entries can be clicked, directing users to the respective dataset. Menus for about, browse and search functions were also included on this page. The search box on the homepage can be used to search any ID or terms in the datasets.
- About page provides general information for TCGA-My and CRC.
- Browse page lists all TCGA-My datasets, four of which are the main datasets and two datasets that contain functional annotation information. These datasets can also be retrieved from the dropdown tab, named Menu, which can be found at the header of each primary page. The datasets on this page are described as follows:
  - Sample dataset: contains genome and metabolome data for CRC. The sample datasets are categorized into gender and ethnicity.
  - Variant dataset: contains variants that were obtained from the 13 samples of genome data for CRC. This dataset is also categorized into chromosomes, DNA regions, tissue occurrence and type of mutations.
  - Gene dataset: contains genes affected by the variants. A list of driver genes can also be obtained.
  - Metabolite dataset: contains metabolites that are profiled in the metabolome data for CRC. This dataset is categorized into class and regulation.
  - Gene ontology dataset: contains GOs information (biological process, molecular function and cellular component) for variant genes in CRC.
  - Pathway dataset: contains pathways information for genes and metabolites of CRC.
- Search page allows two search options, i.e., simple search and variant advanced search. Simple search serves a similar function to the search box on the homepage and the header of the primary pages. Advanced search allows the users to find variant(s) with a combination of different keywords. Users can conduct a quick search for the variants from a certain sample that are linked to a specific driver gene.
- Help page provides an entity-relationship diagram and the table information deposited in this database. The entity-relationship diagram shows the relationship between datasets stored in this database. Table information defines all terms used in TCGA-My. Additionally, this page also provides the contact details for questions or invitations to collaborate.

Each entry in TCGA-My provides a brief description. For instance, if a user searches for one of the patient ID, for example, C187, it will redirect the user to the description page for C187. Four tabs for the mutations/variants, Circos, genes and metabolites identified from C187 data will appear. If a user clicks on one of the entries in the gene tab, for example, gene of ACACA, a description of ACACA will appear and display five tabs for sample, GOs, variants, KEGG and PANTHER pathways. All datasets in TCGA-My are freely downloadable in comma-separated values (CSV) format by clicking the “Download CSV” button located at the top-right of each table.

## 4. Discussion

### 4.1. Strength of TCGA-My

TCGA-My is the first database that houses genome and metabolome data for Malaysian CRC patients. Given the high number of patients and deaths caused by CRC in Malaysia [1], this effort is vital for collecting and collating omics data for in-depth studies that will lead to the development of precision medicine in Malaysia. Details for each patient, such as gender, age, ethnicity, diagnosis information, anatomical location and cancer stage are provided along with other important information to facilitate a reliable prognosis, accurate diagnosis and effective treatment of cancer [36,37].

TCGA-My also contains lists of variants, related genes and metabolites, which are useful for researchers investigating and characterizing the molecules responsible for CRC in specific conditions, such as gender, age, ethnicity, anatomical location or cancer stage. In addition, the decision to treat CRC and other cancers relies on many factors, and each patient has a different condition, so one treatment is ineffective for all conditions. Precision medicine or personalized medicine benefits from omics data and it can provide customized treatment based on the patient’s molecular signatures that will be most effective. Such molecular datasets are crucial for finding potential biomarkers and signatures for CRC that can be used to design the most effective therapy and toward precision medicine [38]. 

TCGA-My is an integrated functional database that contains relevant biological information for genomics and metabolomics data for CRC with links to external databases to ease users into exploring the genes and metabolites. For example, users can find further information about a particular gene by clicking on the GeneCards or UniProt symbol. Further information about GOs and pathways can also be found by clicking on the identifier for the Gene Ontology Consortium [21], KEGG [17] and PANTHER [27] databases, and a window will automatically appear in your browser. In this way, the user is redirected from TCGA-My to a particular database and avoids manual browsing of the original database when searching for a gene of interest. 

This database is systematically organized and provides a dynamic web interface. The users can find all information for each entry on a single page in a separate Table For example, the sample entry provides available information about the patients. All variants, related genes and metabolites can be found on a similar page in different tabs. The genomic data is displayed using a Circos plot to help visualization of the mutation that occurred in a particular genome. Related information on a gene entry can also be found on a similar page with different tabs.

TCGA-My was developed with the aim of providing a platform for a Malaysian cancer consortium, where a CRC dataset was used to provide a model for CRC database development that will be extended gradually. It will also guide the choice of data/samples that will be needed to ensure a comprehensive repository and provide accessible datasets for the epidemiological study of CRC.

### 4.2. Weaknesses of TCGA-My and Future Perspectives

One of the weaknesses of TCGA-My is the absence of another two omics datasets, which are transcriptomics and proteomics. Integrating all omics data for CRC, i.e., genomics, transcriptomics, proteomics and metabolomics, will provide clearer insights into the molecular mechanisms of CRC [39]. Currently, TCGA-My only provides datasets for two major ethnicities in Malaysia, Malay and Chinese, and none for Indians and other minor ethnicities in Malaysia. Nevertheless, the Chinese have been shown to have the highest CRC incidence rate in Malaysia, followed by the Malays [4]. Additionally, there are no datasets available for a younger generation with CRC. TCGA-My provides datasets for ages 41–82 years, but according to the descriptive statistics from the Malaysia Cancer Patient Registry-Colorectal Cancer, CRC incidence rises with age [4]. In addition, early onset of CRC occurs at an age of more than 50 years old in many countries, such as the United States, Canada and Australia [1]. Finally, the Circos plots in TCGA-My are static, making it difficult for users trying to identify the location of particular mutations that occurred in a sample.

Hence, it will be very important to periodically update the datasets in TCGA-My and further improve the interface in order to provide a comprehensive and convenient resource for other researchers to utilize. 

### 4.3. Example of Applications

The first example concerns a molecular cancer researcher who wants to search for a specific gene of interest. Starting with the browse menu, a list of genes with known variants can be obtained, and the relevant information, such as the functional annotation of the molecule and patient statistics, can be retrieved. The users can download the datasets for more advanced analysis as per their intention.

The second example concerns bioinformaticians or biostatisticians interested in conducting big data analysis. With 1.5 million variants that can be downloaded from TCGA-My, there are several analyses that can be conducted, such as advanced data analytics in the search for a correlation between the DNA region and nucleotide changes in the variants. One may also deploy machine learning algorithms, building a model of the relationship between the variants and other columns in the table. Due to clear and unique labelling, this can be deployed easily. 

The last example concerns the data owner. The TCGA-My database provides easy retrieval of the variants and pathways for a patient for future reference or research. The robust architecture of the TCGA-My database also ensures future data can be uploaded smoothly and requires minimal storage and computational resources due to the normalization steps that were deployed during data processing. 

## 5. Conclusions

With rapid advancements in sequencing technologies, new sets of omics data will expand the size of current datasets in TCGA-My. Thus, this database will be periodically updated to ensure it is always up to date. It is essential to provide a specific Malaysian CRC database to ensure the information is accessible by local and global CRC researchers and clinicians for quick and easy reference for further investigation. This database will serve as a systematic and comprehensive omics resource that can be used to search for potential CRC biomarkers for developing improved prognostics, diagnostics, and treatment for CRC.

## Figures and Tables

**Table 1:** Number of entries in the datasets of TCGA-My.

Dataset	Number of Entries
Sample	50
Variant	1,517,841
COSMIC	1113
dbSNP	291,397
Gene	23,695
PDB	4420
RefSeq ncRNA	2637
UniProt	17,910
Metabolite	89,256
Pathway	344
KEGG	186
PANTHER	158
Gene ontology	17,459

**Table 2:** Patients details.

Patient	Gender	Age	Ethnicity	Diagnosis	Anatomical Location	Stage	
						TNM	Dukes
C187	Male	63	Malay	Well differentiated adenocarcinoma	Rectosigmoid	pT3 N2 MX	C2
C330	Male	71	Chinese	Well differentiated adenocarcinoma	Sigmoid colon	T3 N0 MX	B2
C404	Male	68	Chinese	Well differentiated adenocarcinoma	Rectum	pT3 pN1a MX	-
					Sessile polyp in ascending colon	pT1	A
C414	Male	76	Malay	Well differentiated adenocarcinoma (WHO Grade 1)	Sigmoid colon	pT3 pN1b pMX	C
C449	Male	65	Malay	Moderately differentiated adenocarcinoma	Rectosigmoid colon	pT3 N2 MX	C
C476	Male	72	Chinese	Well differentiated adenocarcinoma.	Recto-sigmoidectomy	pT4a N1 MX	
C194	Female	70	Malay	Well differentiated adenocarcinoma	Sigmoid colon	-	B
C273	Female	73	Malay	Moderately differentiated adenocarcinoma	Rectosigmoid colon	pT1 N0 MX	A
C373	Female	74	Chinese	Moderately differentiated adenocarcinoma	Anterior resection specimen	T2 N0 MX	B
C388	Female	65	Chinese	Moderately differentiated adenocarcinoma	Anterior resection specimen	pT2 pN1 pMx	C
C398	Female	71	Chinese	Moderately differentiated adenocarcinoma.	Sigmoid colon with bladder	pT4 N1 MX	C
C467	Female	65	Malay	Well differentiated adenocarcinoma	Rectum	T4b N1b pMX	C
C474	Female	79	Malay	Well-differentiated adenocarcinoma	Left hemicolectomy	pT3 N0 MX	B1

Note: p indicates the pathological state has been examined for the respective component of the TNM staging system.

**Table 3:** TNM and Dukes staging systems.

Staging System	Component		Explanation
TNM	Primary Tumor (T)	T1	Tumor invades submucosa.
		T2	Tumor invades muscularis propria.
		T3	Tumor invades into the subserosa or perirectal tissues via muscularis propria.
		T4	Tumor has spread to other organs or structures directly and/or the visceral peritoneum.
		T4a	The tumor has expanded into the surface of the visceral peritoneum, where it has penetrated all layers of the colon.
		T4b	The tumor has spread to other organs or structures or has attached itself to them.
	Regional lymph node (N)	N0	Negative regional lymph node metastases.
		N1	Metastases in one to three regional lymph nodes.
		N1a	Tumor cells have been detected in one regional lymph node.
		N1b	Tumor cells have been detected in two or three regional lymph nodes.
		N2	Metastases in four or more regional lymph nodes.
	Distant metastases (M)	MX	Distant metastases could not be assessed.
Dukes	A		Tumor limited to the submucosa.
	B		Tumor grows through the colon wall into muscular layers, no lymph nodes involved
	B1		Into but not through the muscularis propria, nodes not involved.
	B2		Through the muscularis propria, nodes not involved.
	C		Lymph node involved.
	C2		Through the muscularis propria with nodes involved.

**Table 4:** List of DNA regions for the variants listed in TCGA-My.

DNA Region	Number of Variants	Description
Intergenic	926,482	Variant overlaps in intergenic region.
Intronic	409,632	Variant overlaps in intronic region.
Non-coding RNA, intronic	84,913	Non-coding transcript variant overlaps with one of the transcripts in the intronic region.
Exonic	8381	Variant overlaps in exonic region.
Upstream	8855	Variant overlaps a 1-kb region upstream of the transcription start site.
Downstream	9116	Variant overlaps a 1-kb region downstream of the transcription termination site.
UTR3	8603	Variant overlap in 3′ untranslated region.
Upstream, downstream	922	Variant overlaps in both upstream and downstream regions.
UTR5	1176	Variant overlaps in 5′ untranslated region.
Splicing	108	Variant overlaps in splice region.
Non-coding RNA, splicing	34	Non-coding transcript variant overlaps with one of the transcripts in the splice region.
Exonic, splicing	2	Variant overlaps in both exonic and splice regions.

**Table 5:** Type of mutations identified for the variants listed in TCGA-My.

Type of Mutations	Number of Variants	Description
Nonsynonymous SNV	3922	A single nucleotide change that alters an amino acid of a protein.
Frameshift insertion	510	Insertion of one or more nucleotides that shifts the codon reading frame.
Frameshift deletion	917	Deletion of one or more nucleotides that shifts the codon reading frame.
Stop-gain	271	Mutations caused by nonsynonymous SNV, frameshift insertion and frameshift deletion that leads to the gain of a stop codon.
Stop-loss	9	Mutations caused by nonsynonymous SNV, frameshift insertion and frameshift deletion that leads to the loss of a stop codon.
Non-frameshift deletion	587	Deletion of a set of nucleotides divisible by three that may not shift a reading frame.
Synonymous SNV	2226	A change of a single nucleotide that retains an amino acid of a protein.
Non-frameshift insertion	153	Insertion of a set of nucleotides divisible by three that may not shift a reading frame.
Unknown	223	Unknown mutation.

**Table 6:** Number of variant genes in genome sample.

Patient	Number of Genes	Number of Driver Genes
C187	11,988	6
C194	11,644	7
C273	11,837	5
C373	11,951	11
C404	13,188	6
C414	12,446	9
C449	13,888	5
C474	23,213	12
C330	11,989	2
C388	11,515	8
C398	11,763	2
C467	12,489	7
C476	11,666	3

## Data Availability

Data is accessible in TCGA-My database.
